# Blood gas phenotyping and tracheal intubation timing in adult in-hospital cardiac arrest: a retrospective cohort study

**DOI:** 10.1038/s41598-021-89920-y

**Published:** 2021-05-18

**Authors:** Chih-Hung Wang, Meng-Che Wu, Cheng-Yi Wu, Chien-Hua Huang, Min-Shan Tsai, Tsung-Chien Lu, Eric Chou, Yen-Wen Wu, Wei-Tien Chang, Wen-Jone Chen

**Affiliations:** 1 Department of Emergency Medicine, National Taiwan University Hospital, No.7, Zhongshan S. Rd., Zhongzheng Dist., Taipei City, 100 Taiwan (R.O.C.); 2 Department of Emergency Medicine, College of Medicine, National Taiwan University, Taipei, Taiwan; 3 Department of Emergency Medicine, Baylor Scott & White All Saints Medical Center, Fort Worth, TX USA; 4 Departments of Internal Medicine and Nuclear Medicine, National Taiwan University Hospital and National Taiwan University College of Medicine, Taipei, Taiwan; 5 Department of Nuclear Medicine and Cardiology Division of Cardiovascular Medical Center, Far Eastern Memorial Hospital, New Taipei City, Taiwan; 6 National Yang-Ming University School of Medicine, Taipei, Taiwan; 7 Division of Cardiology, Department of Internal Medicine, National Taiwan University College of Medicine and Hospital, Taipei, Taiwan

**Keywords:** Cardiology, Diseases

## Abstract

To investigate whether the optimal time to tracheal intubation (TTI) during cardiopulmonary resuscitation would differ by different blood gas phenotypes. Adult patients experiencing in-hospital cardiac arrest (IHCA) from 2006 to 2015 were retrospectively screened. Early intra-arrest blood gas analysis, performed within 10 min of resuscitation, was used to define different phenotypes. In total, 567 patients were included. Non-severe acidosis (pH≧7.15) was associated with favourable neurological outcome (odds ratio [OR]: 4.60, 95% confidence interval [CI] 1.63–12.95; *p* value = 0.004) and survival (OR: 3.25, 95% CI 1.72–6.15; *p* value < 0.001) in the multivariable logistic regression analyses. In the interaction analysis, normal blood gas phenotype (pH: 7.35–7.45, PCO_2_: 35–45 mm Hg, HCO_3_^−^ level: 22–26 mmol/L) × TTI ≦ 6.3 min (OR: 20.40, 95% CI 2.53–164.75; *p* value = 0.005) and non-severe acidosis × TTI ≦ 6.3 min (OR: 3.35, 95% CI 1.00–11.23; *p* value = 0.05) were associated with neurological recovery while metabolic acidosis × TTI ≦ 5.7 min (OR: 3.63, 95% CI 1.36–9.67; *p* value = 0.01) and hypercapnic acidosis × TTI ≦ 10.4 min (OR: 2.27, 95% CI 1.20–4.28; *p* value = 0.01) were associated with survival. Intra-arrest blood gas analysis may help guide TTI during for patients with IHCA.

## Introduction

Approximately 209,000 patients experience in-hospital cardiac arrest (IHCA) in the United States annually^1^. Twenty-four percent of patients with IHCA survive to hospital discharge; among these patients, 14% sustain significant neurological disability^1^.

When treating cardiac arrest, point-of-care blood testing may yield important diagnostic information and guide therapeutic management during cardiopulmonary resuscitation (CPR). For example, the diagnosis of hyperkalaemia could enable the clinicians to rapidly administer potassium-reducing agents to reverse this life-threatening electrolyte abnormality during CPR^2^. However, clinical data on point-of-care blood gas analysis during CPR are limited.

For epidemiological and research purposes, aetiologies of out-of-hospital cardiac arrest are usually categorised into medical and non-medical causes based solely on contextual data without the incorporation of diagnostic testing^3^. Thus, substantial heterogeneity exists between patients even if they are classified within the same category. In scenarios such as prehospital CPR, this crude classification system may be necessary; nonetheless, for IHCA resuscitation, a more elaborate categorization may facilitate the application of appropriate therapeutics. For example, studies^4,5^ indicated that tracheal intubation during CPR may cause harm for patients without respiratory failure prior to IHCA but may not do so for patients with prior respiratory failure.

In this study, we first attempted to investigate whether intra-arrest blood gas analysis could help classify IHCA patients into distinct phenotypes with different prognoses; second, we attempted to investigate whether the optimal timing of tracheal intubation would differ according to different phenotypes. Besides traditionally defined blood gas phenotypes, we would also attempt to identify new phenotypes which may influence the optimal timing of tracheal intubation.

## Materials and methods

### Setting

We used previously established IHCA database for analysis^6,7^. Briefly, the patient data were collected retrospectively at the National Taiwan University Hospital (NTUH). As a tertiary medical centre, NTUH has 2600 beds, including 220 beds in intensive care units (ICUs). This study was performed in accordance with the Declaration of Helsinki amendments. The NTUH Research Ethics Committee approved this study (reference number: 201805098RINC) and waived the requirement for informed consent because of the retrospective and non-interventional nature. In NTUH, a code team is activated whenever an IHCA event occurs in the general wards. A code team consists of a senior resident, several junior residents, a respiratory therapist, a head nurse and several ICU nurses. For IHCA occurring in the ICUs, CPR is performed by the ICU staff without activating a code team. Resuscitation was performed according to recommendations of CPR guidelines^8,9^. In NTUH, point-of-care blood gas analysers were deployed in every ward floor, including floor of general wards and ICUs. Physicians were instructed to obtain blood samples as soon as possible for blood gas analysis in order to identify potential causes leading to IHCA. There were no supra-glottic airways available for IHCA resuscitation in NTUH.

### Participants

Patients experiencing IHCA at NTUH between 2006 and 2015 were screened. Patients fulfilling the following criteria were included for analysis: (1) age above 18 years, (2) chest compressions performed for ≥ 2 min, (3) absence of a do-not-resuscitate order before CPR, (4) early intra-arrest blood gas analysis with available blood pH, partial pressure of carbon dioxide (PCO_2_)_,_ and bicarbonate (HCO_3_^-^) level data and (5) tracheal intubation during CPR. Tracheal intubation included endotracheal intubation, tracheostomy and cricothyroidotomy. If a single patient experienced IHCA events more than once during the hospitalisation, only the first event was analysed. Trauma-related arrest was excluded. The number of patients fulfilling the above criteria during the study period determined the sample size.

### Data collection and outcome measures

The following information was extracted for each patient: age, sex, comorbidities, variables recommended by the Utstein template^10^, early intra-arrest blood gas analysis data and interventions performed at the time of IHCA and after sustained return of spontaneous circulation (ROSC). Sustained ROSC was defined as ROSC lasting consecutively for at least 20 min. Time to intubation was defined as the time interval between the initiation of chest compression to the completion of tracheal intubation. Duration of CPR was recorded as the time interval between the initiation of chest compression until termination of CPR, either due to sustained ROSC or due to declaration of death.

Early intra-arrest blood gas analysis was defined as the first available blood gas data measured within 10 min of initiating CPR, which was usually obtained in the beginning of CPR. The sample for blood gas analysis could be obtained from arterial or venous sources, which could not be verified retrospectively. Blood pH, PCO_2_ and HCO_3_^-^ were measured using point-of-care blood gas analysers. Patients were classified into 5 traditionally defined blood gas phenotypes based on a combination of pH, PCO_2_ and HCO_3_^−^ levels^11,12^: (1) normal (pH: 7.35–7.45, PCO_2_: 35–45 mm Hg, HCO_3_^-^ level: 22–26 mmol/L), (2) non-acidosis, except normal (pH≧7.35, but not belonging to normal), (3) hypercapnic acidosis (pH < 7.35, PCO_2_ > 45 mm Hg, HCO_3_^−^ level > 22 mmol/L), (4) metabolic acidosis (pH < 7.35, PCO_2_ ≦ 45 mm Hg, HCO_3_^−^ level ≦ 22 mmol/L), (5) mixed acidosis (pH < 7.35, but not belonging to hypercapnic or metabolic acidosis).

Favourable neurological status at hospital discharge was selected as the primary outcome, which was defined as one or two points on the Cerebral Performance Category scale^13^. Survival at hospital discharge was selected as the secondary outcome.

### Statistical analysis

We used R 3.3.1 software (R Foundation for Statistical Computing, Vienna, Austria) to analyse data. Categorical variables are presented as counts with proportions, and continuous variables are presented as medians with interquartile ranges. Categorical variables were examined by Chi-squared test while continuous variables were compared by Wilcoxon’s rank-sum test. A two-tailed *p* value < 0.05 was considered significant.

We calculated the odds ratio (OR) as the outcome measure. We conducted univariate and multivariable logistic regression analyses to investigate the associations between variables of interest and outcomes. We placed all available independent variables in the regression model for selection, irrespective of whether they were considered as significant in univariate analyses. We employed generalised additive models (GAMs)^14^ to explore non-linear effects of all continuous variables on outcomes and to identify the optimal cut-off points to transform these continuous variables into binary ones, which would also be tested in the regression analyses. Furthermore, the cut-off points identified for pH, PCO_2_ and HCO_3_^-^ would be used to define new phenotypes. Because the blood gas analysis data, including pH, PCO_2_ and HCO_3_^-^, were used to define different phenotypes, they were not included as independent variables during the model-fitting process. We developed the final regression model by stepwise variable selection procedure with iterations between the forward and backward steps. We defined the significance levels for entry and to stay at 0.15 to avoid exclusion of potential variables. We determined the final regression model by excluding non-significant variables sequentially until all regression coefficients were significant.

In the primary or secondary model, the multivariable analysis was intended to identify the phenotypes associated with primary or secondary outcome, respectively. In the interaction analysis, the interaction between each phenotype and time to intubation was assessed during the model-fitting process. We assessed the goodness-of-fit of the regression models by *c* statistics, the adjusted generalised *R*^2^ and the Hosmer–Lemeshow goodness-of-fit test.

We performed a sensitivity analysis to assess the influence of patients with missing blood gas analysis data. We performed the multiple imputation procedure to impute the missing data. The packages of Amelia and Zelig were employed. We used predictors altogether to impute the missing values. Ten datasets were imputed, and each dataset was used individually to fit the final regression model obtained in the primary analysis. The ten fitted results were then pooled into a single coefficient in the regression model.

## Results

As shown in Supplemental Figure 1, there were 1698 adult non-trauma patients of IHCA who received CPR for ≥ 2 min at NTUH between 2006 and 2015. Of these, 599 patients were excluded because of the lack of early intra-arrest blood gas analysis, including 573 patients not having blood gas analysis and 26 patients having missing blood gas analysis data. In the remaining 1099 patients, 311 patients received intubation before IHCA and 221 patients did not receive intubation during CPR; therefore, a total of 567 patients were included in the analysis. The comparisons between patients with and without blood gas analysis data and among patients stratified by the timing of tracheal intubation were demonstrated in Supplemental Tables 1–4.

The features of included patients are presented in Tables 1 and 2. Median patient age was 69.9 years. Median CPR duration was 31.0 min and median time to intubation was 7.0 min. Median pH, PCO_2_, and HCO_3_^−^ levels were 7.2, 54.3 mmHg and 19.6 mmol/L, respectively. Hypercapnic acidosis was the most dominant traditional blood gas phenotypes (214 patients, 37.7%). Only 66 patients (11.6%) survived to hospital discharge; of these, 30 patients (5.3%) demonstrated favourable neurological status.

**Table 1:** Baseline characteristics of study patients.

Variables	All patients (n = 567)	Patients with favourable neurological outcome at hospital discharge (n = 30)	Patients without favourable neurological outcome at hospital discharge (n = 537)	*p* value	Odds ratio (95% confidence interval)
Age, years (IQRSD^a^)	69.9 (57.7–79.8)	65.2 (52.1–73.4)	70.5 (58.2–80.4)	0.02	0.98 (0.96–1.00)
Male, n (%)	342 (60.3)	24 (80.0)	318 (59.2)	0.023	2.75 (1.11–6.85)
**Comorbidities, n (%)**					
Heart failure, this admission	95 (16.8)	6 (20.0)	89 (16.6)	0.5862	1.26 (0.50–3.17)
Heart failure, prior admission	88 (15.5)	5 (16.7)	83 (15.5)	0.7780	1.09 (0.41–2.94)
Myocardial infarction, this admission	61 (10.8)	6 (20.0)	55 (10.2)	0.172	2.19 (0.86–5.59)
Myocardial infarction, prior admission	22 (3.9)	2 (6.7)	20 (3.7)	0.303	1.85 (0.41–8.30)
Arrhythmia	103 (18.2)	2 (6.7)	101 (18.8)	0.194	0.31 (0.07–1.32)
Hypotension	92 (16.2)	6 (20.0)	86 (16.0)	0.5561	1.31 (0.52–3.30)
Respiratory insufficiency	355 (62.6)	19 (63.3)	336 (62.6)	> 0.99	1.03 (0.48–2.22)
Renal insufficiency	209 (36.9)	13 (43.3)	196 (36.5)	0.3844	1.33 (0.63–2.80)
Hepatic insufficiency	102 (18.0)	6 (20.0)	96 (17.9)	0.7681	1.15 (0.46–2.89)
Metabolic or electrolyte abnormality	81 (14.3)	4 (13.3)	77 (14.3)	> 0.99	0.92 (0.31–2.71)
Diabetes mellitus	197 (34.7)	6 (20.0)	191 (35.6)	0.0911	0.45 (0.18–1.13)
Baseline evidence of motor, cognitive, or functional deficits	178 (31.4)	7 (23.3)	171 (31.8)	0.4442	0.65 (0.27–1.55)
Acute stroke	28 (4.9)	1 (3.3)	27 (5.0)	> 0.99	0.65 (0.09–4.96)
Favourable neurological status 24 h before cardiac arrest	335 (59.1)	22 (73.3)	313 (58.3)	0.0813	1.97 (0.86–4.50)
Pneumonia	151 (26.6)	4 (13.3)	147 (27.4)	0.1613	0.41 (0.14–1.19)
Bacteraemia	41 (7.2)	0 (0)	41 (7.6)	0.186	0.20 (0.01–3.27)
Cirrhosis	44 (7.8)	1 (3.3)	43 (8.0)	0.702	0.40 (0.05–2.98)
Chronic obstructive pulmonary disease	32 (5.6)	2 (6.7)	30 (5.6)	0.618	1.21 (0.27–5.31)
Dialysis	89 (15.7)	6 (20.0)	83 (15.5)	0.415	1.37 (0.54–3.45)
Metastatic cancer or any blood-borne malignancy	129 (22.8)	2 (6.7)	127 (23.6)	0.034	0.23 (0.05–0.98)
Charlson comorbidity index (IQRSD)	2.9 (1–42.3)	2.3 (1–32.0)	3.0 (1–42.3)	0.11	0.86 (0.71–1.03)

^a^IQRSD, interquartile rangesstandard deviation.

**Table 2:** Features, interventions and outcomes of cardiac arrest events.

Variables	All patients (n = 567)	Patients with favourable neurological outcome at hospital discharge (n = 30)	Patients without favourable neurological outcome at hospital discharge (n = 537)	*p* value	Odds ratio (95% confidence interval)
Arrest at night, n (%)	225 (39.7)	9 (30.0)	216 (40.2)	0.2834	0.64 (0.29–1.42)
Arrest on weekend, n (%)	150 (26.5)	5 (16.7)	145 (27.0)	0.2529	0.54 (0.20–1.44)
Arrest location, n (%)				0.079	0.66 (0.33–1.32)
Intensive care unit	130 (22.9)	11 (36.7)	119 (22.2)		
General ward	396 (69.8)	16 (53.3)	380 (70.8)		
Others	41 (7.2)	3 (10.0)	38 (7.1)		
Witnessed arrest, n (%)	312 (55.0)	20 (66.7)	292 (54.4)	0.216	1.68 (0.77–3.65)
Monitored status, n (%)	254 (44.8)	18 (60.0)	236 (43.9)	0.1209	1.91 (0.90–4.05)
Shockable rhythm, n (%)	58 (10.2)	7 (23.3)	51 (9.5)	0.023	2.90 (1.19–7.09)
**Critical care interventions in place at time of arrest, n (%)**					
Non-invasive positive-pressure ventilation	92 (16.2)	4 (13.3)	88 (16.4)	0.7880	0.79 (0.27–2.31)
Antiarrhythmics	53 (9.3)	4 (13.3)	49 (9.1)	0.4451	1.53 (0.51–4.57)
Vasopressors	168 (29.6)	11 (36.7)	157 (29.2)	0.3841	1.40 (0.65–3.01)
Dialysis	25 (4.4)	2 (6.7)	23 (4.3)	0.379	1.60 (0.36–7.11)
Pulmonary artery catheter	1 (0.2)	0 (0)	1 (0.2)	> 0.99	5.86 (0.23–146.95)
Intra-aortic balloon pumping	6 (1.1)	1 (3.3)	5 (0.9)	0.238	3.67 (0.42–32.44)
CPR^a^ duration, min (IQRSD^b^)	31.040.6 (17.0–53.039.3)	15.016.7 (7.0–22.011.7)	342.0 (18.0–55.040.0)	< 0.001	0.93 (0.91–0.96)
Time to intubation, min (IQRSD)	7.08.9 (4.0–12.08.6)	4.55.4 (2.0–7.04.8)	7.09.1 (4.0–12.08.7)	0.005	0.91 (0.84–0.98)
**Intra-arrest blood gas analysis**					
Blood pH (IQRSD)	7.2 (7.0–7.30.2)	7.3 (7.2–7.30.2)	7.21 (7.0–7.30.2)	< 0.001	24.87 (3.31–186.81)
PCO_2_,^c^ mmHg (IQRSD)	54.363.7 (39.6–77.940.2)	41.29.8 (35.7–57.724.6)	54.864.4 (39.7–78.240.8)	0.02	0.98 (0.97–1.00)
HCO_3_^−^, mmol/L (IQRSD)	19.620.5 (14.3–24.79.8)	20.61.4 (15.9–24.87.8)	19.620.4 (14.1–24.69.9)	0.41	1.01 (0.98–1.04)
**Traditional blood gas phenotype**					
Normal	13 (2.3)	2 (6.7)	11 (2.0)	0.115	3.42 (0.72–16.16)
Non-acidosis, except normal	67 (11.8)	5 (16.7)	62 (11.5)	0.4238	1.53 (0.57–4.15)
Hypercapnic acidosis	214 (37.7)	10 (33.3)	204 (38.0)	0.750	0.82 (0.37–1.78)
Metabolic acidosis	119 (21.0)	10 (33.3)	114 (21.2)	0.2617	1.96 (0.89–4.32)
Mixed acidosis	154 (27.2)	3 (10)	151 (28.1)	0.023	0.28 (0.08–0.95)
New blood gas phenotype		30	537		
Non-severe acidosis	300 (52.9)	25 (8.3)	275 (51.2)	< 0.001	4.76 (1.80–12.62)
Severe hypercapnic acidosis	138 (24.3)	3 (2.2)	135 (25.1)	0.068	0.33 (0.10–1.11)
Severe metabolic acidosis	77 (13.6)	2 (2.6)	75 (14.0)	0.4341	0.44 (0.10–1.89)
Severe mixed acidosis	52 (9.2)	0 (0)	52 (9.7)	0.130	0.15 (0.01–2.52)
**Post-ROSC**^**d**^** interventions, n (%)**					
Extracorporeal membrane oxygenation	52 (9.2)	6 (20.0)	46 (8.6)	0.065	2.67 (1.04–6.86)
Targeted temperature management	6 (1.1)	2 (6.7)	4 (0.7)	0.034	9.52 (1.67–54.20)
Percutaneous coronary intervention	24 (4.2)	7 (23.3)	17 (3.2)	< 0.001	9.31 (3.51–24.66)
Sustained ROSC, n (%)	344 (60.7)	30 (100)	314 (58.5)	< 0.001	43.35 (2.64–712.69)
Survival to hospital discharge, n (%)	66 (11.6)	30 (100)	36 (6.7)	< 0.001	838.12 (50.23–13,985.57)

^a^CPR, cardiopulmonary resuscitation.

^b^SDIQR, interquartile rangesstandard deviation.

^c^PCO_2_, partial pressure of carbon dioxide.

^d^ROSC, return of spontaneous circulation.

**Figure 1:**
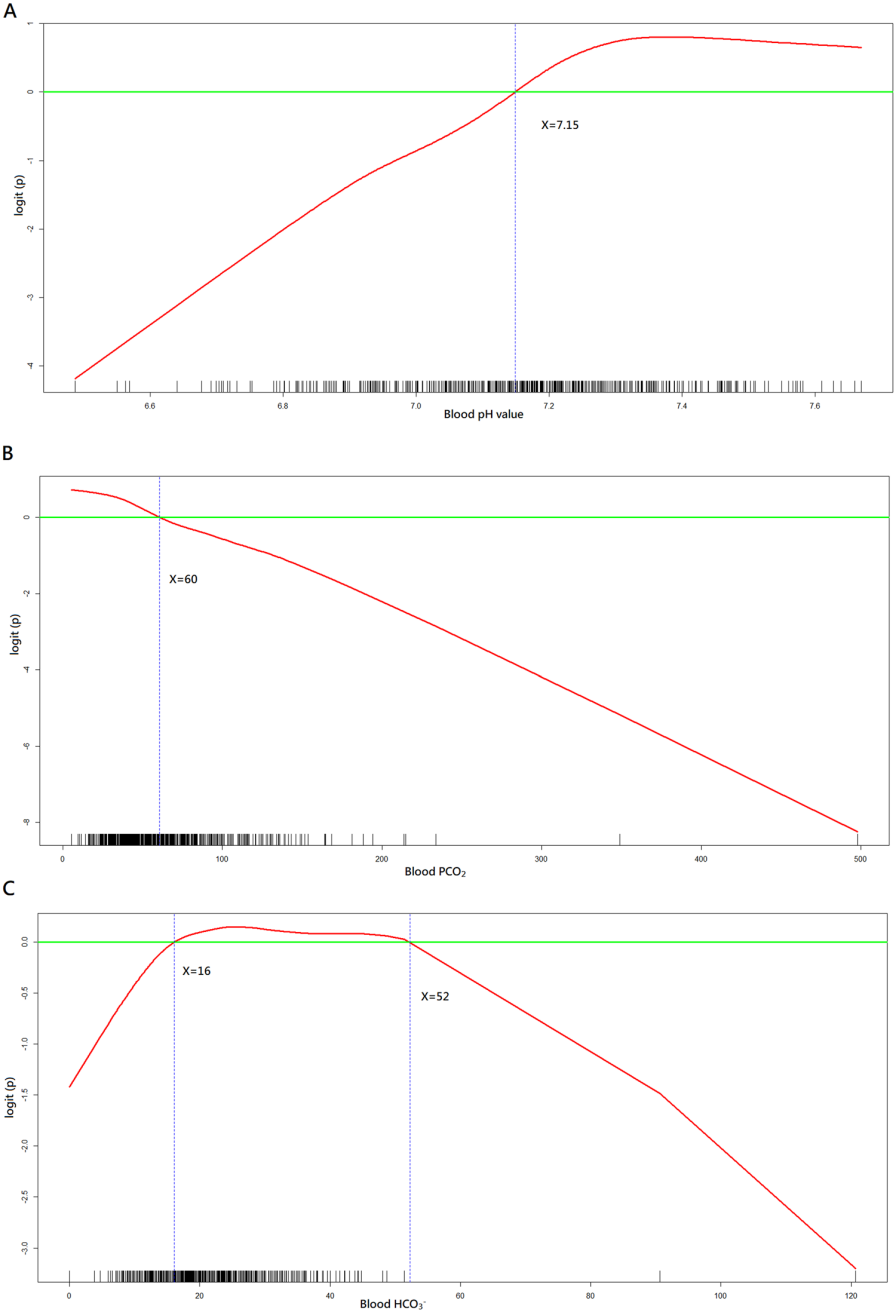
Generalised additive model plots for nonparametric modelling of the respective effect of (**A**) blood pH, (**B**) PCO_2_, and (**C**) HCO_3_^−^ on the logit of probability for favourable neurological outcome at hospital discharge. R Core Team (2019). R: A language and environment for statistical computing. R Foundation for Statistical Computing, Vienna, Austria. URL http://www.R-project.org/.

The GAM plots illustrated the association of logit (p), where p represented the probability for the primary outcome, with pH, PCO_2_, and HCO_3_^−^ levels, respectively (Fig. 1). If logit (p) was greater than zero, the odds for achieving favourable neurological outcome would be greater than one. Therefore, pH of 7.15, PCO_2_ of 60 mmHg and HCO_3_^-^ level of 16 mmol/L were selected as cut-off points to define new blood gas phenotypes, as follows: (1) non-severe acidosis (pH ≧ 7.15), (2) severe hypercapnic acidosis (pH < 7.15, PCO_2_ > 60 mm Hg, HCO_3_^−^ level > 16 mmol/L), (3) severe metabolic acidosis (pH < 7.15, PCO_2_  ≦  60 mm Hg, HCO_3_^−^ level  ≦  16 mmol/L), (4) severe mixed acidosis (pH < 7.15, but not belonging to severe hypercapnic or metabolic acidosis). Both the new and traditional blood gas phenotypes were placed in the variable list for variable selection during the process of building primary and secondary models. Similarly, time to intubation was transformed into a binary variable based on GAM plots (Supplemental Figs. 2–5) and tested in the analysis.

For the primary outcome (Table 3), in the primary model, non-severe acidosis was positively associated with favourable neurological outcome (OR: 4.60, 95% confidence interval [CI] 1.63–12.95; *p* value = 0.004). The baseline characteristics and IHCA features stratified by severe acidosis are presented in Supplemental Tables 5–6. In the interaction analysis, both normal blood gas phenotype × time to intubation ≦ 6.3 min (OR: 20.40, 95% CI 2.53–164.75; *p* value = 0.005) and non-severe acidosis × time to intubation ≦ 6.3 min (OR: 3.35, 95% CI 1.00–11.23; *p* value = 0.05) were positively associated with favourable neurological outcome.

**Table 3:** Multiple logistic regression model with favourable neurological outcome at hospital discharge as the dependent variable.

Independent variable^a^	Odds ratio	95% confidence interval	*p* value
**Primary model**			
CPR^b^ duration	0.93	0.90–0.96	< 0.001
Post-ROSC^c^ percutaneous coronary intervention	5.81	1.94–17.39	0.002
Non-severe acidosis	4.60	1.63–12.95	0.004
Age	0.97	0.94–0.99	0.01
Male	2.79	1.04–7.44	0.04
**Primary model with interaction terms**			
CPR duration	0.93	0.90–0.97	< 0.001
Non-severe acidosis	7.31	2.38–22.48	< 0.001
Post-ROSC percutaneous coronary intervention	8.14	2.26–29.30	0.001
Normal blood gas phenotype × time to intubation ≦ 6.3 (min)	20.40	2.53–164.75	0.005
Age between 28 and 74 (years)	4.88	1.60–14.92	0.005
Diabetes mellitus	0.21	0.07–0.66	0.008
Male	3.05	1.09–8.50	0.03
Arrest on weekend	0.27	0.08–0.91	0.04
Non-severe acidosis × time to intubation ≦ 6.3 (min)	3.35	1.00–11.23	0.05

Primary model: goodness-of-fit assessment: n = 567, adjusted generalized *R*^*2*^ = 0.32,estimated area under the receiver operating characteristic curve = 0.89, and Hosmer and Lemeshow goodness-of-fit Chi-Squared test *p* = 0.51; Primary model with interaction terms: goodness-of-fit assessment: n = 567, adjusted generalized *R*^*2*^ = 0.42, estimated area under the receiver operating characteristic curve = 0.91, and Hosmer and Lemeshow goodness-of-fit Chi-Squared test *p* = 0.46.

^a^The display of independent variables is arranged in order of *p* value.

^b^CPR, cardiopulmonary resuscitation.

^c^ROSC, return of spontaneous circulation.

For the secondary outcome (Table 4), in the secondary model, non-severe acidosis was positively associated with survival (OR: 3.25, 95% CI 1.72–6.15; *p* value < 0.001). In the interaction analysis, both metabolic acidosis × time to intubation  ≦  5.7 min (OR: 3.63, 95% CI 1.36–9.67; *p* value = 0.01) and hypercapnic acidosis × time to intubation  ≦  10.4 min (OR: 2.27, 95% CI 1.20–4.28; *p* value = 0.01) were positively associated with survival.

**Table 4:** Multiple logistic regression model with survival at hospital discharge as the dependent variable.

Independent variable^a^	Odds ratio	95% confidence interval	*p* value
**Secondary model**			
CPR^b^ duration	0.94	0.92–0.96	< 0.001
Non-severe acidosis	3.25	1.72–6.15	< 0.001
Charlson comorbidity index	0.82	0.71–0.94	0.005
Post-ROSC^c^ extracorporeal membrane oxygenation	3.15	1.37–7.25	0.007
Male	1.98	1.07–3.64	0.03
**Secondary model with interaction terms**			
CPR duration	0.94	0.92–0.96	< 0.001
Non-severe acidosis	3.18	1.66–6.08	< 0.001
Post-ROSC extracorporeal membrane oxygenation	3.50	1.50–8.15	0.004
Charlson comorbidity index	0.82	0.71–0.95	0.006
Metabolic acidosis × time to intubation ≦ 5.7 (min)	3.63	1.36–9.67	0.01
Hypercapnic acidosis × time to intubation ≦ 10.4 (min)	2.27	1.20–4.28	0.01
Male	2.02	1.08–3.76	0.03

Secondary model: goodness-of-fit assessment: n = 567, adjusted generalized *R*^*2*^ = 0.29,estimated area under the receiver operating characteristic curve = 0.83, and Hosmer and Lemeshow goodness-of-fit Chi-Squared test *p* < 0.001; Secondary model with interaction terms: goodness-of-fit assessment: n = 567, adjusted generalized *R*^*2*^ = 0.32, estimated area under the receiver operating characteristic curve = 0.84, and Hosmer and Lemeshow goodness-of-fit Chi-Squared test *p* < 0.001.

^a^The display of independent variables is arranged in order of *p* value.

^b^CPR, cardiopulmonary resuscitation.

^c^ROSC, return of spontaneous circulation.

In the sensitivity analysis, we imputed the data for the 26 patients who had missing blood gas analysis data and were excluded from the primary analysis. As shown in Supplemental Table 7, when imputed data were used to fit the primary model with interaction terms, the effect estimates of the included variables were similar to those of the original model (Table 3).

## Discussion

### Main findings

The results suggest that intra-arrest blood gas analysis might be used to classify IHCA patients into distinct phenotypes with different prognoses and responses to intervention. Non-severe acidosis, one of the phenotypes, was found to be positively associated with better neurological and survival outcomes. In the interaction analysis, for patients with non-severe acidosis, shorter time to intubation was associated with favourable neurological outcome, while for patients with metabolic or hypercapnic acidosis, shorter time to intubation was associated with improved survival. The results of interaction analysis suggested that the effects of time to intubation may differ between different phenotypes. Phenotyping by blood gas analysis may enable patient-tailored intervention during CPR.

### Phenotyping by blood gas analysis and tracheal intubation

The most common feature used to phenotype patients during CPR may be the initial arrest rhythm, i.e. shockable versus non-shockable rhythm^8,9^, which significantly influences outcomes and necessitates prompt defibrillation for the former. However, beyond this, there are few reported features able to phenotype patients and essentially, all patients receive universal or one-size-fits-all managements^8,9^. The inability to phenotype patients may weaken the benefits of a certain intervention in some subgroups, or even cause harm, which might explain the abundant neutral results from clinical trials on cardiac arrest^15^. Point-of-care blood gas analysis had the potential to phenotype patients during CPR and guide corresponding treatment. Our results demonstrated a significant benefit of shorter time to intubation for patients with non-severe acidosis and normal blood gas phenotype regarding favourable neurological recovery. The normal blood gas phenotype was actually a subgroup of non-severe acidosis. Because the effects of shorter time to intubation were substantially greater for normal blood gas phenotype than non-severe acidosis (OR: 20.40 versus 3.35), the variables of shorter time to intubation with respective phenotypes were present in the primary model with interaction terms simultaneously. Earlier intubation during CPR may facilitate better control of ventilation and oxygenation^16^, resulting in better outcomes.

In our study, the benefits of shorter time to intubation were only observed in patients of non-severe acidosis, suggesting that the tracheal intubation may only be effective for patients with higher “resuscitability,” i.e. patients with higher chances of achieving favourable neurological or survival outcomes. Spindelboeck et al^17^ once reported that as high as 98% of OHCA patients had intra-arrest pH < 7.35. The mix of patients with high or low “resuscitability” may have thus nullified the potential benefits of intubation on long-term neurological and survival outcomes in previous studies^18^. In contrast to OHCA resuscitated in the out-of-hospital environment, IHCA patients usually could be attended earlier with more healthcare personnel involved. Therefore, it may be more likely for in-hospital code teams to conduct point-of-care blood gas analysis in determining whether early tracheal intubation should be performed during CPR for IHCA patients.

Most studies^4,5,19^ used large registry data to investigate the effect of tracheal intubation. Despite the increased statistical power with the larger study population^4,5,19^, the patients could only be classified under broad medical categories, such as shockable or non-shockable rhythm. In contrast, the phenotypes classified by blood gas analysis might be more reflective of the real-time pathophysiological states and be more appropriate to guide the corresponding treatment. The confounding effects of the different blood gas phenotypes, such as non-severe acidosis, could not accounted for in these studies^4,5,19^, which might lead to the heterogeneous results.

### Clinical applications

Because our study was based on a highly selected cohort (approximately 33% [567/1698] of screened patients underwent blood gas analysis and intubation), the results should be further examined before clinical use. For example, the blood pH value used to define severe acidosis may need more patients to be validated. However, in the secondary model, we did find that for patients with traditionally defined hypercapnic or metabolic acidosis, shorter time to intubation was associated with better survival, which may be more clinically relevant. In most previous studies, point-of-care intra-arrest blood gas analysis data, such as pH^20–22^, PCO_2_^23^, base excess^24^, and lactate^25^ levels, were used as single prognosticators to predict outcomes following CPR. For clinicians, it may be difficult to remember these individually identified predictors and relevant cut-off points in predicting poor outcomes. The use of common phenotypes, such as hypercapnic or metabolic acidosis, may be more familiar and intuitive for clinicians to react with corresponding interventions.

### Study limitations

First, because of the nature of a retrospective study design, we could only establish an association, rather than a causal relationship, between independent variables and outcomes. Additionally, the effects of unmeasured confounding factors may also introduce bias into the analysis. For example, the indications for blood gas analysis and tracheal intubation could not be retrospectively obtained and adjusted for in the multivariable analysis. Second, blood sampling during CPR was technically difficult and therefore, it was difficult to verify whether blood sample was drawn from an artery or a vein source. Although venous pH may be consistent with arterial pH^26,27^, discrepancies between arterial and venous PCO_2_ levels have been reported^28^. This could only be resolved by a prospective study adopting a certified method to obtain arterial blood gas. Third, we did not record the timing and the amount of sodium bicarbonate administered during CPR. The changes in blood pH, PCO_2_ or HCO_3_^-^ caused by administration of sodium bicarbonate^29^ may lead to misclassification bias, which may nullify the association between some blood gas phenotypes and outcomes. However, since the administration of sodium bicarbonate may not significantly improve or worsen the resuscitation outcomes^29^, the effect of misclassification bias could probably be mitigated to some extent. For example, the significant association between non-severe acidosis and neurological outcomes was still identified in the regression analysis. Fourth, despite that we had accounted for the confounding effects of CPR duration and time to intubation, the resuscitation time bias^30^ may still be concerning since those patients without intubation during CPR were excluded from the analysis. Fifth, the exact timing of obtaining blood gas samples was not recorded in the previously established IHCA database^6,7^ and therefore, we could not verify whether the timing of obtaining blood gas samples was prior to the timing of intubation or administration of sodium bicarbonate. Nonetheless, since the clinicians in NTUH were instructed to obtain blood gas analysis as soon as possible in the beginning of CPR, the interval between the timing of blood gas analysis and intubation or administration of sodium bicarbonate may be quite small (maximum: 10 min), which may not cause significant changes in blood gas analysis data. Finally, during the study period between 2006 and 2015, the guidelines^31,32^ did not recommend the optimal timing of tracheal intubation. The timing of tracheal intubation might be influenced by other unmeasured confounders, such as clinicians’ experience in tracheal intubation. While early tracheal intubation was associated with better outcomes in some phenotypes, the generalizability of this practice should be further examined in a prospective study since this study was based on a highly selected cohort. What was emphasized in the current study was that blood gas analysis could help classify patients into different phenotypes that were associated with outcome and with the effect of a certain intervention. However, the phenotypes may be simplified, and the cut-off points used may not be accurate. Therefore, results of the current investigation are best viewed as a proof-of-concept rather than a definite conclusion, which should be further examined in a prospective study.

## Conclusions

Intra-arrest blood gas analysis may assist in phenotyping patients with IHCA. Non-severe acidosis was associated with better neurological and survival outcomes. For patients with non-severe acidosis, early tracheal intubation was associated with favourable neurological outcome, while for patients with metabolic or hypercapnic acidosis, early tracheal intubation was associated with improved survival.

## Supplementary Information

Supplementary Information 1.

Supplementary Information 2.

Supplementary Information 3.

Supplementary Information 4.

Supplementary Information 5.

Supplementary Information 6.

Supplementary Information 7.

Supplementary Information 8.

Supplementary Information 9.

Supplementary Information 10.

Supplementary Information 11.

Supplementary Information 12.

Supplementary Information 13.

## Data Availability

The data that support the findings of this study are available on request from the corresponding author, Wen-Jone Chen.
