# Supplementary material for: Blood gas phenotyping and tracheal intubation timing in adult in-hospital cardiac arrest: a retrospective cohort study
Source: Sci Rep. 2021 May 18;11:10480. doi: 10.1038/s41598-021-89920-y (PMC8131623; doi:10.1038/s41598-021-89920-y)
Supplement: Supplementary file 1 — Supplementary Information 1. [file 41598_2021_89920_MOESM1_ESM.docx]

**Supplemental Figure 1**. Patient selection algorithm. CPR, cardiopulmonary resuscitation; IHCA, in-hospital cardiac arrest; ROSC, return of spontaneous circulation.

**Supplemental Figure 2**. Generalised additive model plots for nonparametric modelling of the effect of time to intubation on the logit of probability for favourable neurological outcome at hospital discharge for patients of normal blood gas phenotype. Each dot on x-axis represents each patient data. R Core Team (2019). R: A language and environment for statistical computing. R Foundation for Statistical Computing, Vienna, Austria. URL http://www.R-project.org/.

**Supplemental Figure 3**. Generalised additive model plots for nonparametric modelling of the effect of time to intubation on the logit of probability for favourable neurological outcome at hospital discharge for patients of non-severe acidosis. Each dot on x-axis represents each patient data. R Core Team (2019). R: A language and environment for statistical computing. R Foundation for Statistical Computing, Vienna, Austria. URL http://www.R-project.org/.

**Supplemental Figure 4**. Generalised additive model plots for nonparametric modelling of the effect of time to intubation on the logit of probability for survival at hospital discharge for patients of hypercapnic acidosis. Each dot on x-axis represents each patient data. R Core Team (2019). R: A language and environment for statistical computing. R Foundation for Statistical Computing, Vienna, Austria. URL http://www.R-project.org/.

**Supplemental Figure 5**. Generalised additive model plots for nonparametric modelling of the effect of time to intubation on the logit of probability for survival at hospital discharge for patients of metabolic acidosis. Each dot on x-axis represents each patient data. R Core Team (2019). R: A language and environment for statistical computing. R Foundation for Statistical Computing, Vienna, Austria. URL http://www.R-project.org/.
