# Supplementary material for: Blood gas phenotyping and tracheal intubation timing in adult in-hospital cardiac arrest: a retrospective cohort study
Source: Sci Rep. 2021 May 18;11:10480. doi: 10.1038/s41598-021-89920-y (PMC8131623; doi:10.1038/s41598-021-89920-y)
Supplement: Supplementary file 7 — Supplementary Information 7. [file 41598_2021_89920_MOESM7_ESM.docx]

**Blood Gas Phenotyping and Tracheal Intubation Timing in Adult In-hospital Cardiac Arrest: A Retrospective Cohort Study**

Chih-Hung Wang, MD, PhD; Meng-Che Wu, MD; Cheng-Yi Wu, MD; Chien-Hua Huang, MD, PhD; Min-Shan Tsai, MD, PhD; Tsung-Chien Lu, MD, PhD; Eric Chou, MD; Yen-Wen Wu, MD, PhD; Wei-Tien Chang, MD, PhD; Wen-Jone Chen, MD, PhD

Supplemental Table 1. Comparison of baseline characteristics between patients with and without blood gas data

| Variables | All screened patients  (n = 1698) | Patients with blood gas data (n = 1099) | Patients without blood gas data (n = 599) | *p*-value |
| --- | --- | --- | --- | --- |
| Age, years (SD^a^) | 65.0 (16.8) | 66 (16.6) | 63 (17.0) | 0.001 |
| Male, n (%) | 1063 (61.0) | 673 (61.2) | 363 (60.6) | 0.84 |
| Comorbidities, n (%) |  |  |  |  |
| Heart failure, this admission | 328 (19.3) | 210 (19.1) | 118 (19.7) | 0.80 |
| Heart failure, prior admission | 274 (16.1) | 174 (15.8) | 100 (16.7) | 0.68 |
| Myocardial infarction, this admission | 216 (12.7) | 133 (12.1) | 83 (13.9) | 0.32 |
| Myocardial infarction, prior admission | 73 (4.3) | 43 (3.9) | 30 (5.0) | 0.32 |
| Arrhythmia | 306 (18.0) | 203 (18.5) | 103 (17.2) | 0.55 |
| Hypotension | 417 (24.6) | 265 (24.1) | 152 (25.4) | 0.60 |
| Respiratory insufficiency | 1224 (72.1) | 795 (72.3) | 429 (71.6) | 0.78 |
| Renal insufficiency | 698 (41.1) | 458 (41.7) | 240 (40.1) | 0.54 |
| Hepatic insufficiency | 305 (18.0) | 193 (17.6) | 112 (18.7) | 0.60 |
| Metabolic or electrolyte  abnormality | 284 (16.7) | 190 (17.3) | 94 (15.7) | 0.41 |
| Diabetes mellitus | 556 (32.7) | 368 (33.5) | 188 (31.4) | 0.39 |
| Baseline evidence of motor, cognitive, or functional deficits | 538 (31.7) | 339 (30.8) | 199 (33.2) | 0.33 |
| Acute stroke | 75 (4.4) | 48 (4.4) | 27 (4.5) | 0.90 |
| Favourable neurological status 24 h before cardiac arrest | 737 (43.4) | 478 (43.5) | 259 (43.2) | 0.96 |
| Pneumonia | 540 (31.8) | 346 (31.5) | 194 (32.4) | 0.70 |
| Bacteraemia | 144 (8.5) | 93 (8.5) | 51 (8.5) | >0.99 |
| Cirrhosis | 116 (6.8) | 72 (6.6) | 44 (7.3) | 0.55 |
| Chronic obstructive pulmonary disease | 89 (5.2) | 62 (5.6) | 27 (4.5) | 0.36 |
| Dialysis | 294 (17.3) | 199 (18.1) | 95 (15.9) | 0.25 |
| Metastatic cancer or any blood-borne malignancy | 388 (22.9) | 247 (22.5) | 141 (23.5) | 0.63 |
| Charlson comorbidity index (SD) | 2.9 (2.2) | 3.0 (2.2) | 2.8 (2.2) | 0.08 |

Categorical variables were examined by Fisher’s exact test while continuous variables were compared by Wilcoxon’s rank-sum test.

^a^SD, standard deviation
