# Supplementary material for: Blood gas phenotyping and tracheal intubation timing in adult in-hospital cardiac arrest: a retrospective cohort study
Source: Sci Rep. 2021 May 18;11:10480. doi: 10.1038/s41598-021-89920-y (PMC8131623; doi:10.1038/s41598-021-89920-y)
Supplement: Supplementary file 8 — Supplementary Information 8. [file 41598_2021_89920_MOESM8_ESM.docx]

**Blood Gas Phenotyping and Tracheal Intubation Timing in Adult In-hospital Cardiac Arrest: A Retrospective Cohort Study**

Chih-Hung Wang, MD, PhD; Meng-Che Wu, MD; Cheng-Yi Wu, MD; Chien-Hua Huang, MD, PhD; Min-Shan Tsai, MD, PhD; Tsung-Chien Lu, MD, PhD; Eric Chou, MD; Yen-Wen Wu, MD, PhD; Wei-Tien Chang, MD, PhD; Wen-Jone Chen, MD, PhD

Supplemental Table 2. Comparison of features, interventions and outcomes of cardiac arrest events between patients with and without blood gas data

| Variables | | All screened patients  (n = 1698) | | Patients with blood gas data (n = 1099) | | Patients without blood gas data (n = 599) | *p*-value |
| --- | --- | --- | --- | --- | --- | --- | --- |
| Arrest at night, n (%) | | 564 (33.2) | | 397 (36.1) | | 167 (27.9) | <0.001 |
| Arrest on weekend, n (%) | | 485 (28.6) | | 307 (27.9) | | 178 (29.7) | 0.46 |
| Arrest location, n (%) | |  | |  | |  | 0.15 |
| Intensive care unit | | 779 (45.9) | | 495 (45.0) | | 284 (47.4) |  |
| General ward | | 811 (47.8) | | 541 (49.2) | | 270 (45.1) |  |
| Others | | 108 (6.4) | | 63 (5.7) | | 45 (7.5) |  |
| Witnessed arrest, n (%) | | 1202 (70.8) | | 768 (69.9) | | 434 (72.5) | 0.29 |
| Monitored status, n (%) | | 1057 (6.2) | | 677 (61.6) | | 380 (63.4) | 0.46 |
| Shockable rhythm, n (%) | | 266 (15.7) | | 160 (14.6) | | 106 (17.7) | 0.09 |
| Critical care interventions in place at time of arrest, n (%) | |  | |  | |  |  |
| Non-invasive positive-pressure ventilation | | 410 (24.1) | | 268 (24.4) | | 142 (23.7) | 0.77 |
| Antiarrhythmics | | 197 (11.6) | | 125 (11.4) | | 72 (12.0) | 0.69 |
| Vasopressors | | 760 (44.8) | | 472 (42.9) | | 288 (48.1) | 0.05 |
| Dialysis | | 123 (7.2) | | 86 (7.8) | | 37 (6.2) | 0.24 |
| Pulmonary artery catheter | | 14 (0.8) | | 6 (0.5) | | 8 (1.3) | 0.10 |
| Intra-aortic balloon pumping | | 19 (1.1) | | 11 (1.0) | | 8 (1.3) | 0.63 |
| CPR^a^ duration, min (SD^b^) | | 33.1 (33.6) | | 36.4 (37.1) | | 27.1 (24.1) | <0.001 |
| Time to intubation, min (SD) | | 9.6 (9.4) (n=843) | | 9.5 (9.3) (n=595) | | 9.8 (9.7) (n=248) | 0.93 |
| Post-ROSC^c^ interventions, n (%) | |  | |  | |  |  |
| Extracorporeal membrane oxygenation | | 139 (8.2) | | 90 (8.2) | | 49 (8.2) | >0.99 |
| Targeted temperature management | | 13 (0.8) | | 8 (0.7) | | 5 (0.8) | 0.78 |
| Percutaneous coronary intervention | 80 (4.7) | | 40 (3.6) | | 40 (6.7) | | 0.006 |
| Sustained ROSC, n (%) | 970 (57.1) | | 621 (56.5) | | 349 (58.3) | | 0.50 |
| Survival to hospital discharge, n (%) | 240 (14.1) | | 138 (12.6) | | 102 (17.0) | | 0.01 |
| Favourable neurological outcome at hospital discharge, n (%) | 124 (7.3) | | 67 (6.1) | | 57 (9.5) | | 0.01 |

Categorical variables were examined by Fisher’s exact test while continuous variables were compared by Wilcoxon’s rank-sum test.

^a^CPR, cardiopulmonary resuscitation

^b^SD, standard deviation

^c^ROSC, return of spontaneous circulation
