# Supplementary material for: Blood gas phenotyping and tracheal intubation timing in adult in-hospital cardiac arrest: a retrospective cohort study
Source: Sci Rep. 2021 May 18;11:10480. doi: 10.1038/s41598-021-89920-y (PMC8131623; doi:10.1038/s41598-021-89920-y)
Supplement: Supplementary file 9 — Supplementary Information 9. [file 41598_2021_89920_MOESM9_ESM.docx]

**Blood Gas Phenotyping and Tracheal Intubation Timing in Adult In-hospital Cardiac Arrest: A Retrospective Cohort Study**

Chih-Hung Wang, MD, PhD; Meng-Che Wu, MD; Cheng-Yi Wu, MD; Chien-Hua Huang, MD, PhD; Min-Shan Tsai, MD, PhD; Tsung-Chien Lu, MD, PhD; Eric Chou, MD; Yen-Wen Wu, MD, PhD; Wei-Tien Chang, MD, PhD; Wen-Jone Chen, MD, PhD

Supplemental Table 3. Comparison of baseline characteristics of patients with blood gas data stratified by timing of tracheal intubation

| Variables | Patients receiving tracheal intubation before cardiopulmonary resuscitation  (n = 311) | Patients receiving tracheal intubation during cardiopulmonary resuscitation (n = 567) | Patients not receiving tracheal intubation during cardiopulmonary resuscitation (n = 221) | *p*-value |
| --- | --- | --- | --- | --- |
| Age, years (SD^a^) | 64.9 (17.4) | 67.4 (16.0) | 64.0(16.5) | 0.02 |
| Male, n (%) | 213 (68.5) | 342 (60.3) | 118 (53.4) | 0.51 |
| Comorbidities, n (%) |  |  |  |  |
| Heart failure, this admission | 66 (21.2) | 95 (16.8) | 49 (22.2) | 0.05 |
| Heart failure, prior admission | 57 (18.3) | 88 (15.5) | 29 (13.1) | 0.73 |
| Myocardial infarction, this admission | 40 (12.9) | 61 (10.8) | 33 (14.9) | 0.09 |
| Myocardial infarction, prior admission | 20 (6.4) | 22 (3.9) | 11 (5.0) | 0.34 |
| Arrhythmia | 57 (18.3) | 103 (18.2) | 42 (19.0) | 0.14 |
| Hypotension | 108 (34.7) | 92 (16.2) | 64 (29.0) | <0.001 |
| Respiratory insufficiency | 290 (93.2) | 355 (62.6) | 148 (67.0) | <0.001 |
| Renal insufficiency | 154 (49.5) | 209 (36.9) | 93 (42.1) | 0.006 |
| Hepatic insufficiency | 62 (19.9) | 102 (18.0) | 29 (13.1) | 0.48 |
| Metabolic or electrolyte  abnormality | 57 (18.3) | 81 (14.3) | 52 (23.5) | <0.001 |
| Diabetes mellitus | 100 (32.2) | 197 (34.7) | 71 (32.1) | 0.25 |
| Baseline evidence of motor, cognitive, or functional deficits | 82 (26.4) | 178 (31.4) | 78 (35.3) | 0.001 |
| Acute stroke | 12 (3.9) | 28 (4.9) | 8 (3.6) | 0.61 |
| Favourable neurological status 24 h before cardiac arrest | 62 (19.9) | 335 (59.1) | 81 (36.7) | <0.001 |
| Pneumonia | 133 (42.8) | 151 (26.6) | 61 (27.6) | <0.001 |
| Bacteraemia | 37 (11.9) | 41 (7.2) | 14 (6.3) | 0.10 |
| Cirrhosis | 21 (6.8) | 44 (7.8) | 7 (3.2) | 0.11 |
| Chronic obstructive pulmonary disease | 20 (6.4) | 32 (5.6) | 10 (4.5) | 0.90 |
| Dialysis | 69 (22.2) | 89 (15.7) | 40 (18.1) | 0.12 |
| Metastatic cancer or any blood-borne malignancy | 79 (25.4) | 129 (22.8) | 40 (18.1) | 0.65 |
| Charlson comorbidity index (SD) | 3.1 (2.3) | 2.9 (2.3) | 2.8 (2.1) | 0.17 |

Categorical variables were examined by Chi-squared test while continuous variables were compared by one-way ANOVA test.

^a^SD, standard deviation
