# Supplementary material for: Blood gas phenotyping and tracheal intubation timing in adult in-hospital cardiac arrest: a retrospective cohort study
Source: Sci Rep. 2021 May 18;11:10480. doi: 10.1038/s41598-021-89920-y (PMC8131623; doi:10.1038/s41598-021-89920-y)
Supplement: Supplementary file 10 — Supplementary Information 10. [file 41598_2021_89920_MOESM10_ESM.docx]

**Blood Gas Phenotyping and Tracheal Intubation Timing in Adult In-hospital Cardiac Arrest: A Retrospective Cohort Study**

Chih-Hung Wang, MD, PhD; Meng-Che Wu, MD; Cheng-Yi Wu, MD; Chien-Hua Huang, MD, PhD; Min-Shan Tsai, MD, PhD; Tsung-Chien Lu, MD, PhD; Eric Chou, MD; Yen-Wen Wu, MD, PhD; Wei-Tien Chang, MD, PhD; Wen-Jone Chen, MD, PhD

Supplemental Table 4. Features, interventions and outcomes of cardiac arrest events of patients with blood gas data stratified by timing of tracheal intubation

| Variables | | Patients receiving tracheal intubation before cardiopulmonary resuscitation  (n = 311) | | Patients receiving tracheal intubation during cardiopulmonary resuscitation (n = 567) | | Patients not receiving tracheal intubation during cardiopulmonary resuscitation (n = 221) | *p*-value |
| --- | --- | --- | --- | --- | --- | --- | --- |
| Arrest at night, n (%) | | 108 (34.7) | | 225 (39.7) | | 64 (29.0) | 0.04 |
| Arrest on weekend, n (%) | | 92 (29.6) | | 150 (26.5) | | 65 (29.4) | 0.22 |
| Arrest location, n (%) | |  | |  | |  | <0.001 |
| Intensive care unit | | 261 (83.9) | | 130 (22.9) | | 105 (47.5) |  |
| General ward | | 63 (20.3) | | 396 (69.8) | | 81 (36.7) |  |
| Others | | 10 (3.2) | | 41 (7.2) | | 12 (5.4) |  |
| Witnessed arrest, n (%) | | 308 (99.0) | | 312 (55.0) | | 148 (67.0) | <0.001 |
| Monitored status, n (%) | | 292 (93.9) | | 254 (44.8) | | 131 (59.3) | <0.001 |
| Shockable rhythm, n (%) | | 55 (17.7) | | 58 (10.2) | | 47 (21.3) | <0.001 |
| Critical care interventions in place at time of arrest, n (%) | |  | |  | |  |  |
| Non-invasive positive-pressure ventilation | | 118 (37.9) | | 92 (16.2) | | 60 (27.1) | <0.001 |
| Antiarrhythmics | | 52 (16.7) | | 53 (9.3) | | 12 (5.4) | 0.02 |
| Vasopressors | | 239 (76.8) | | 168 (29.6) | | 68 (30.8) | <0.001 |
| Dialysis | | 39 (12.5) | | 25 (4.4) | | 22 (10.0) | <0.001 |
| Pulmonary artery catheter | | 1 (0.3) | | 1 (0.2) | | 4 (1.8) | 0.008 |
| Intra-aortic balloon pumping | | 1 (0.3) | | 6 (1.1) | | 4 (1.8) | 0.15 |
| CPR^a^ duration, min (SD^b^) | | 36.0 (37.7) | | 40.6 (39.3) | | 24.9 (25.5) | <0.001 |
| Intra-arrest blood gas analysis | |  | |  | |  |  |
| Blood pH (SD) | | 7.3 (0.2) | | 7.2 (0.2) | | 7.2 (0.2) | <0.001 |
| PCO_2_,^c^ mmHg (SD) | | 52.8 (36.0) | | 63.7 (40.2) | | 58.1 (43.7) | <0.001 |
| HCO_3-_, mmol/L (SD) | | 22.8 (16.2) | | 20.5 (9.8) | | 22.9 (18.3) | 0.02 |
| Post-ROSC^d^ interventions, n (%) | |  | |  | |  |  |
| Extracorporeal membrane oxygenation | | 18 (5.8) | | 52 (9.2) | | 20 (9.0) | 0.08 |
| Targeted temperature management | | 1 (0.3) | | 6 (1.1) | | 1 (0.5) | 0.40 |
| Percutaneous coronary intervention | 8 (2.6) | | 24 (4.2) | | 8 (3.6) | | 0.34 |
| Sustained ROSC, n (%) | 155 (49.8) | | 344 (60.7) | | 122 (55.2) | | <0.001 |
| Survival to hospital discharge, n (%) | 33 (10.6) | | 66 (11.6) | | 39 (17.6) | | <0.001 |
| Favourable neurological outcome at hospital discharge, n (%) | 14 (4.5) | | 30 (5.3) | | 23 (10.4) | | 0.001 |

Categorical variables were examined by Chi-squared test while continuous variables were compared by one-way ANOVA test.

^a^CPR, cardiopulmonary resuscitation

^b^SD, standard deviation

^c^PCO_2_, partial pressure of carbon dioxide

^d^ROSC, return of spontaneous circulation
