# Supplementary material for: Blood gas phenotyping and tracheal intubation timing in adult in-hospital cardiac arrest: a retrospective cohort study
Source: Sci Rep. 2021 May 18;11:10480. doi: 10.1038/s41598-021-89920-y (PMC8131623; doi:10.1038/s41598-021-89920-y)
Supplement: Supplementary file 11 — Supplementary Information 11. [file 41598_2021_89920_MOESM11_ESM.docx]

**Blood Gas Phenotyping and Tracheal Intubation Timing in Adult In-hospital Cardiac Arrest: A Retrospective Cohort Study**

Chih-Hung Wang, MD, PhD; Meng-Che Wu, MD; Cheng-Yi Wu, MD; Chien-Hua Huang, MD, PhD; Min-Shan Tsai, MD, PhD; Tsung-Chien Lu, MD, PhD; Eric Chou, MD; Yen-Wen Wu, MD, PhD; Wei-Tien Chang, MD, PhD; Wen-Jone Chen, MD, PhD

Supplemental Table 5. Baseline characteristics of study patients stratified by the severe acidosis

| Variables | All patients  (n = 567) | Patients with non-severe acidosis (n = 300) | Patients with severe acidosis (n = 267) | *p*-value |
| --- | --- | --- | --- | --- |
| Age, years (IQR^a^) | 69.9 (57.7-79.8) | 70.9 (59.3-81.0) | 70.5 (58.2-80.4) | 0.06 |
| Male, n (%) | 342 (60.3) | 187 (62.3) | 581 (15.5) | 0.30 |
| Comorbidities, n (%) |  |  |  |  |
| Heart failure, this admission | 95 (16.8) | 64 (21.3) | 31 (11.6) | 0.002 |
| Heart failure, prior admission | 88 (15.5) | 58 (19.3) | 30 (11.2) | 0.008 |
| Myocardial infarction, this admission | 61 (10.8) | 43 (14.3) | 18 (6.7) | 0.004 |
| Myocardial infarction, prior admission | 22 (3.9) | 16 (5.3) | 6 (2.2) | 0.06 |
| Arrhythmia | 103 (18.2) | 65 (21.7) | 38 (14.2) | 0.02 |
| Hypotension | 92 (16.2) | 37 (13.9) | 55 (18.3) | 0.15 |
| Respiratory insufficiency | 355 (62.6) | 190 (63.3) | 165 (61.8) | 0.71 |
| Renal insufficiency | 209 (36.9) | 118 (39.3) | 91 (34.1) | 0.20 |
| Hepatic insufficiency | 102 (18.0) | 60 (20.0) | 42 (15.7) | 0.19 |
| Metabolic or electrolyte  abnormality | 81 (14.3) | 43 (14.3) | 38 (14.2) | 0.97 |
| Diabetes mellitus | 197 (34.7) | 117 (39.0) | 80 (30.0) | 0.02 |
| Baseline evidence of motor, cognitive, or functional deficits | 178 (31.4) | 96 (32.0) | 82 (30.7) | 0.74 |
| Acute stroke | 28 (4.9) | 18 (6.0) | 10 (3.7) | 0.22 |
| Favourable neurological status 24 h before cardiac arrest | 335 (59.1) | 185 (61.7) | 150 (56.2) | 0.19 |
| Pneumonia | 151 (26.6) | 79 (26.3) | 72 (27.0) | 0.87 |
| Bacteraemia | 41 (7.2) | 18 (6.0) | 23 (8.6) | 0.23 |
| Cirrhosis | 44 (7.8) | 23 (7.7) | 21 (7.9) | 0.93 |
| Chronic obstructive pulmonary disease | 32 (5.6) | 23 (7.7) | 9 (3.4) | 0.03 |
| Dialysis | 89 (15.7) | 53 (17.7) | 36 (13.5) | 0.17 |
| Metastatic cancer or any blood-borne malignancy | 129 (22.8) | 56 (18.7) | 73 (27.3) | 0.01 |
| Charlson comorbidity index (IQR) | 2 (1-4) | 2 (1-4) | 3 (1-4) | 0.61 |

^a^IQR, interquartile ranges
