# Supplementary material for: Blood gas phenotyping and tracheal intubation timing in adult in-hospital cardiac arrest: a retrospective cohort study
Source: Sci Rep. 2021 May 18;11:10480. doi: 10.1038/s41598-021-89920-y (PMC8131623; doi:10.1038/s41598-021-89920-y)
Supplement: Supplementary file 12 — Supplementary Information 12. [file 41598_2021_89920_MOESM12_ESM.docx]

**Blood Gas Phenotyping and Tracheal Intubation Timing in Adult In-hospital Cardiac Arrest: A Retrospective Cohort Study**

Chih-Hung Wang, MD, PhD; Meng-Che Wu, MD; Cheng-Yi Wu, MD; Chien-Hua Huang, MD, PhD; Min-Shan Tsai, MD, PhD; Tsung-Chien Lu, MD, PhD; Eric Chou, MD; Yen-Wen Wu, MD, PhD; Wei-Tien Chang, MD, PhD; Wen-Jone Chen, MD, PhD

Supplemental Table 6. Features, interventions and outcomes of cardiac arrest events stratified by the severe acidosis

| Variables | | All patients  (n = 567) | | Patients with non-severe acidosis (n = 300) | | Patients with severe acidosis (n = 267) | *p*-value |
| --- | --- | --- | --- | --- | --- | --- | --- |
| Arrest at night, n (%) | | 225 (39.7) | | 107 (35.7) | | 118 (44.2) | 0.04 |
| Arrest on weekend, n (%) | | 150 (26.5) | | 79 (26.3) | | 71 (26.6) | 0.94 |
| Arrest location, n (%) | |  | |  | |  | 0.01 |
| Intensive care unit | | 130 (22.9) | | 83 (27.7) | | 47 (17.6) |  |
| General ward | | 396 (69.8) | | 194 (64.7) | | 202 (75.7) |  |
| Others | | 41 (7.2) | | 23 (7.7) | | 18 (6.7) |  |
| Witnessed arrest, n (%) | | 312 (55.0) | | 174 (58.0) | | 138 (51.7) | 0.13 |
| Monitored status, n (%) | | 254 (44.8) | | 148 (49.3) | | 106 (39.7) | 0.02 |
| Shockable rhythm, n (%) | | 58 (10.2) | | 46 (15.3) | | 12 (4.5) | <0.001 |
| Critical care interventions in place at time of arrest, n (%) | |  | |  | |  |  |
| Non-invasive positive-pressure ventilation | | 92 (16.2) | | 51 (17.0) | | 41 (15.4) | 0.60 |
| Antiarrhythmics | | 53 (9.3) | | 32 (10.7) | | 21 (7.9) | 0.25 |
| Vasopressors | | 168 (29.6) | | 98 (32.7) | | 70 (26.2) | 0.09 |
| Dialysis | | 25 (4.4) | | 14 (4.7) | | 11 (4.1) | 0.75 |
| Pulmonary artery catheter | | 1 (0.2) | | 0 (0) | | 1 (0.4) | 0.29 |
| Intra-aortic balloon pumping | | 6 (1.1) | | 3 (1.0) | | 3 (1.1) | 0.89 |
| CPR^a^ duration, min (IQR^b^) | | 31.0 (17.0-53.0) | | 30.0 (17.0-50.0) | | 32.0 (18.0-55.0) | 0.22 |
| Time to intubation, min (IQR) | | 7.0 (4.0-12.0) | | 6.0 (3.0-10.0) | | 9.0 (5.0-13.8) | 0.002 |
| Intra-arrest blood gas analysis | |  | |  | |  |  |
| Blood pH (IQR) | | 7.2 (7.0-7.3) | | 7.3 (7.2-7.4) | | 7.0 (6.9-7.1) | <0.001 |
| PCO_2_,^c^ mmHg (IQR) | | 54.3 (39.6-77.9) | | 44.9 (33.2-58.3) | | 73.7 (52.6-98.6) | <0.001 |
| HCO3^-^, mmol/L (IQR) | | 19.6 (14.3-24.7) | | 21.2 (16.2-25.8) | | 18.2 (12.2-23.3) | <0.001 |
| Traditional blood gas phenotype | |  | |  | |  |  |
| Normal | | 13 (2.3) | | 13 (4.3) | | 0 (0) | <0.001 |
| Non-acidosis, except normal | | 67 (11.8) | | 67 (22.3) | | 0 (0) | <0.001 |
| Hypercapnic acidosis | | 214 (37.7) | | 112 (37.3) | | 102 (38.2) | 0.83 |
| Metabolic acidosis | | 119 (21.0) | | 75 (25.0) | | 44 (16.5) | 0.01 |
| Mixed acidosis | | 154 (27.2) | | 33 (11) | | 121 (45.3) | <0.001 |
| New blood gas phenotype | |  | | 30 | | 537 |  |
| Severe hypercapnic acidosis | | 138 (24.3) | | 0 (0) | | 138 (51.7) | <0.001 |
| Severe metabolic acidosis | | 77 (13.6) | | 0 (0) | | 77 (28.8) | <0.001 |
| Severe mixed acidosis | | 52 (9.2) | | 0 (0) | | 52 (19.5) | <0.001 |
| Post-ROSC^d^ interventions, n (%) | |  | |  | |  |  |
| Extracorporeal membrane oxygenation | | 52 (9.2) | | 35 (11.7) | | 17 (6.4) | 0.03 |
| Targeted temperature management | | 6 (1.1) | | 4 (1.3) | | 2 (0.7) | 0.50 |
| Percutaneous coronary intervention | 24 (4.2) | | 17 (5.7) | | 7 (2.6) | | 0.07 |
| Sustained ROSC, n (%) | 344 (60.7) | | 188 (62.7) | | 156 (58.4) | | 0.30 |
| Survival to hospital discharge, n (%) | 66 (11.6) | | 51 (17.0) | | 15 (5.6) | | <0.001 |
| Favourable neurological outcome at hospital discharge, n (%) | 30 (5.3) | | 25 (8.3) | | 5 (1.9) | |  |

^a^CPR, cardiopulmonary resuscitation

^b^IQR, interquartile ranges

^c^PCO_2_, partial pressure of carbon dioxide

^d^ROSC, return of spontaneous circulation
