# Supplementary material for: Blood gas phenotyping and tracheal intubation timing in adult in-hospital cardiac arrest: a retrospective cohort study
Source: Sci Rep. 2021 May 18;11:10480. doi: 10.1038/s41598-021-89920-y (PMC8131623; doi:10.1038/s41598-021-89920-y)
Supplement: Supplementary file 13 — Supplementary Information 13. [file 41598_2021_89920_MOESM13_ESM.docx]

**Blood Gas Phenotyping and Tracheal Intubation Timing in Adult In-hospital Cardiac Arrest: A Retrospective Cohort Study**

Chih-Hung Wang, MD, PhD; Meng-Che Wu, MD; Cheng-Yi Wu, MD; Chien-Hua Huang, MD, PhD; Min-Shan Tsai, MD, PhD; Tsung-Chien Lu, MD, PhD; Eric Chou, MD; Yen-Wen Wu, MD, PhD; Wei-Tien Chang, MD, PhD; Wen-Jone Chen, MD, PhD

Supplemental Table 7. Sensitivity analysis using imputed data to fit the primary model with interaction terms

| Independent variable^a^ | Odds ratio | 95% confidence interval | p value |
| --- | --- | --- | --- |
| *Primary model with interaction terms (models using imputed data)* | | | |
| CPR^b^ duration | 0.92 | 0.91-0.96 | <0.001 |
| Non-severe acidosis | 7.45 | 2.33-23.5 | <0.001 |
| Post-ROSC^c^ percutaneous coronary intervention | 8.47 | 2.75-30.12 | 0.002 |
| Normal blood gas phenotype×time to intubation≦6.3 (min) | 20.51 | 2.79-162.45 | 0.004 |
| Age between 28 and 74 (years) | 4.75 | 1.65-15.03 | 0.006 |
| Diabetes mellitus | 0.19 | 0.05-0.45 | 0.007 |
| Male | 3.13 | 1.12-8.96 | 0.04 |
| Arrest on weekend | 0.35 | 0.09-0.95 | 0.05 |
| Non-severe acidosis×time to intubation≦6.3 (min) | 3.45 | 1.01-12.13 | 0.05 |

^a^ The display of independent variables is arranged in order of *p* value.

^b^ CPR, cardiopulmonary resuscitation

^c^ ROSC, return of spontaneous circulation
